# Reproducibility and Consistency of Methods to Define Hospital-Level Procedural Volume Thresholds for Pancreatectomy

**DOI:** 10.1002/jso.70134

**Published:** 2025-11-16

**Authors:** Kristen N. Kaiser, Alexa J. Hughes, Jeannette W. Chung, Adam S. Wilk, Katie Ross-Driscoll, Rachel E. Patzer, Karl Y. Bilimoria, Ryan J. Ellis

**Affiliations:** 1 Department of Surgery, Indiana School of Medicine, Surgical Outcomes and Quality Improvement Center (SOQIC), Indianapolis, Indiana; 2 Center for Health Services Research, Regenstrief Institute, Indianapolis, Indiana; 3 Division of Transplant Surgery, Department of Surgery, Indiana University School of Medicine, Indianapolis, Indiana; 4 Division of Surgical Oncology, Department of Surgery, Indiana University School of Medicine, Indianapolis, Indiana

**Keywords:** classification and regression trees, cubic splines, optimal cutpoints, pancreatectomy, stratum specific likelihood ratios, volume-outcome relationship

## Abstract

**Introduction:**

Procedural volume thresholds (VTs) for hospital quality reporting rely on expert consensus or analytic methods that may produce inconsistent VTs (e.g. restricted cubic splines (RCS), optimal cutpoints, classification and regression trees (CART), stratum specific likelihood ratios (SSLR)). The objective of this study was to compare variation in hospital-level VTs for pancreatectomy across multiple methodologies.

**Methods:**

Patients undergoing pancreatectomy from 2004 to 2021 were identified using the National Cancer Database. RCS, optimal cutpoints, CART, and SSLR were used to compute VTs based on 90-day mortality. From a single clinical data set, VTs were derived multiple times for each method by varying statistical parameters within each model.

**Results:**

Overall, 61,920 patients underwent pancreatectomy at 982 hospitals. VTs associated with reductions in 90-day mortality ranged from 9.2 to 26.1 cases/year (RCS), 15.7–33.8 cases/year (optimal cutpoints), and 11–18 cases/year (CART), all based on modifiable statistical parameters. SSLR analysis yielded a singular VT of 21 cases/year without variability due to lack of statistical input.

**Conclusion:**

Among 4 common strategies for identifying VT that we studied, SSLR required the fewest assumptions. This may make it ideal for enhancing transparency and standardization in outcomes reporting.

## 1 ∣ Introduction

The association between improved outcomes and higher surgical volumes for complex oncologic procedures is well established [1-8]. Key stakeholders, such as Leapfrog and the American Cancer Society, rely on expert consensus in defining high volume centers [9-11]. Investigators have used a variety of models to characterize how surgical volume and outcomes are related. Considering differences within each of these models themselves, as well as differences in study populations and samples, data-driven approaches have derived a vast range of threshold values across different studies for a given procedure. Consequently, there is significant heterogeneity in the literature regarding what constitutes high volume and the methodology for determining these volume cutoffs remains inconsistent.

Current model-based approaches defining volume thresholds (e.g., cubic splines, optimal cutpoints) within existing literature vary in their underlying assumptions, reflected in their use of predictor variables and various interpretation methods. These assumptions vary from one study and data set to another, contributing to the wide variability in volume thresholds (VTs) derived across studies. For example, for pancreatic resections, proposed high VTs in the literature range from 5 to 125 cases per year [5]. Even when methods are rigorously reported, heterogeneity in VTs render it difficult to translate any derived thresholds into practical quality reports or to standardize analyses of procedural volume and outcomes.

Despite efforts to define VTs, robust thresholds that are consistent across studies remain elusive due to lack of reproducibility in methodologies. Achieving consistency in methodology is critical for establishing actionable thresholds that can drive meaningful policy changes and consistent quality reporting. Desirable attributes of a methodology for deriving volume thresholds for quality reporting analysis of volume and outcomes include that the underlying volume-outcome model is reproducible, that it does not rely on any modifiable parametric input, and that it produces multiple thresholds that can be incorporated into a downstream clinical model that adjusts for patient and hospital characteristics. Therefore, the objectives of this study were to (1) assess variability in hospital-level volume thresholds for pancreatectomy based on modification of model parameters utilizing restricted cubic splines, optimal cutpoints, classification and regression trees, and SSLR methodologies and (2) describe and compare the range of threshold values produced by each model.

## 2 ∣ Methods

### 2.1 ∣ Study Population

This study used the National Cancer Database (NCDB) participant user file (PUF) to identify patients with pancreatic cancer who underwent pancreatectomy from 2004 to 2021. Any patients who underwent pancreatectomy were included in the analysis. Patients were excluded if they underwent local excision. The NCDB is comprised of patients treated at Commission on Cancer (CoC) sites across the United States and includes data on treatment for more than 70% of the invasive cancer diagnoses nationwide [12]. The project was deemed nonhuman subjects research by the Indiana University Institutional Review Board.

Facility volume was calculated as the total number of pancreatectomies performed at each facility divided by the total number of calendar years the facility was in the NCDB with at least one pancreatectomy. All data from this cohort of patients were analyzed to derive volume thresholds associated with a reduction in the risk of 90-day mortality following pancreatectomy based on quantiles, restricted cubic splines, optimal cutpoints, CART and SSLR (Supporting Information Table 1).

### 2.2 ∣ Restricted Cubic Splines

RCS are used to visually and mathematically represent non-linear relationships between an independent and dependent variable. The range of the independent variable is split into segments, at individual “knots”, which define where the relationship is modeled for the best fit. Optimal fit is frequently defined by 3–5 knots [13-15]. Additionally, specific knot locations can be defined within the RCS model, as shown in Supporting Information Table 2. Once knot number and locations are specified, the model generates a smooth, continuous curve. Multiple RCS models were generated, with each model varying in the number and location of knots. The best-fitting model was identified for this study based on Akaike Information Criterion (AIC) and Bayesian Information Criterion (BIC).

Once an RCS model of the relationship between facility volume and outcomes has been estimated, several methodologies can be employed to determine optimal thresholds along the RCS curve. Having identified the best-fitting RCS model, threshold values from that model can be identified in several ways: the elbow method, point closest to (0, 1), Youden index, index of union, and inflection point (second derivative) [16, 17]. The elbow method involves identifying the point of maximal deviation between the RCS curve and a linear line fitted between the curve's endpoints (Supporting Information Figure 1) [17, 18]. Using the receiver operator curve, point closest to (0, 1), Youden index and index of union were all used to find the maximized probability on the curve and subsequently the corresponding hospital volume [16, 19]. The inflection point method calculates the threshold as the point with the maximum absolute value of the second derivative [20]. The volume thresholds derived from these methods were compared within a range defined by the highest and lowest values produced.

### 2.3 ∣ Optimal Cutpoints

“Optimal cutpoints” is a collection of methods for identifying cutpoints of a continuous variable to segment a population into different groups. Optimal cutpoints can be determined using cutpointr, an R (Vienna, Austria) package designed for the interpretation of mathematical models and the determination of a specific “cutpoint” (in this case, volume threshold) based on input data and specific methodologic parameters. The package leverages the Youden Index as its primary metric for identifying optimal cutpoints. Each computation provides a unique method for identifying the cutpoint, and the model supports boot-strapping to optimize results. In the current study, the specific method was varied (e.g. spline, locally estimated scatterplot smoothing [loess], etc) while the number of bootstrap permutations was set to 1000 to ensure robust output.

### 2.4 ∣ Conditional and Regression Trees

An additional methodology employed was based on classification and regression trees (CART) using the rpart package in R. CART is a form of supervised machine learning that partitions data into groups with similar predictive outcomes based on covariates (in this case 90-day mortality) [21]. CART creates unique thresholds based on where a significant break in the data occurs on continuous variables (annual case volume). One input parameter that defines how many breaks or nodes is the complexity parameter. The complexity parameter within the CART was varied to examine the effect of pruning (allowing for more or less covariate splits) on interpretable volume thresholds. The covariates selected were based on availability of variables within the NCDB and included age, sex, Charlson Deyo Score, race & ethnicity, insurance status, pathologic T stage, and facility volume (rounded to nearest integer). Variable importance scores were generated from the CART to quantify the contribution of each predictor to the final model.

### 2.5 ∣ Stratum Specific Likelihood Ratios

The SSLR method creates strata based on likelihood ratios between the independent and dependent variables [22, 23]. For the present study, facility volume was rounded to the nearest whole number to define each stratum (i.e., every integer representing an observed, rounded hospital volume was its own stratum to start the analysis). SSLR was performed using unadjusted likelihood ratios calculated from the distribution of outcomes across volume strata. Multivariable adjustment was not applied, Within each stratum a likelihood ratio was calculated with its variance and the 95% confidence interval (CI) without adjustment for any parameters. Strata with overlapping CIs were iteratively collapsed, producing representative thresholds. The collapse happens starting with the first two thresholds, if CIs overlap, they collapse and a new SSLR is run. The process continues until all volume strata have no overlap in their confidence intervals. These thresholds correspond to volumes associated with precipitous declines in the risk of the primary outcome (90-day mortality). To define a singular VT across the representative strata produced by SSLR, the VT where the SSLR < 1 with no overlap of 1 on the confidence interval was selected [23].

This study was designed and reported in accordance with the Strengthening the Reporting of Observational Studies in Epidemiology (STROBE) guidelines [24]. Missing data were handled using complete case analysis; All methodologies were compared for their VTs produced, the reproducibility, parametric input, and number of thresholds. All analyses were run in R Studio (Vienna, Austria), Stata (College Station, Texas) and Microsoft Excel (Seattle, Washington).

## 3 ∣ Results

**FIGURE 1 ∣:**
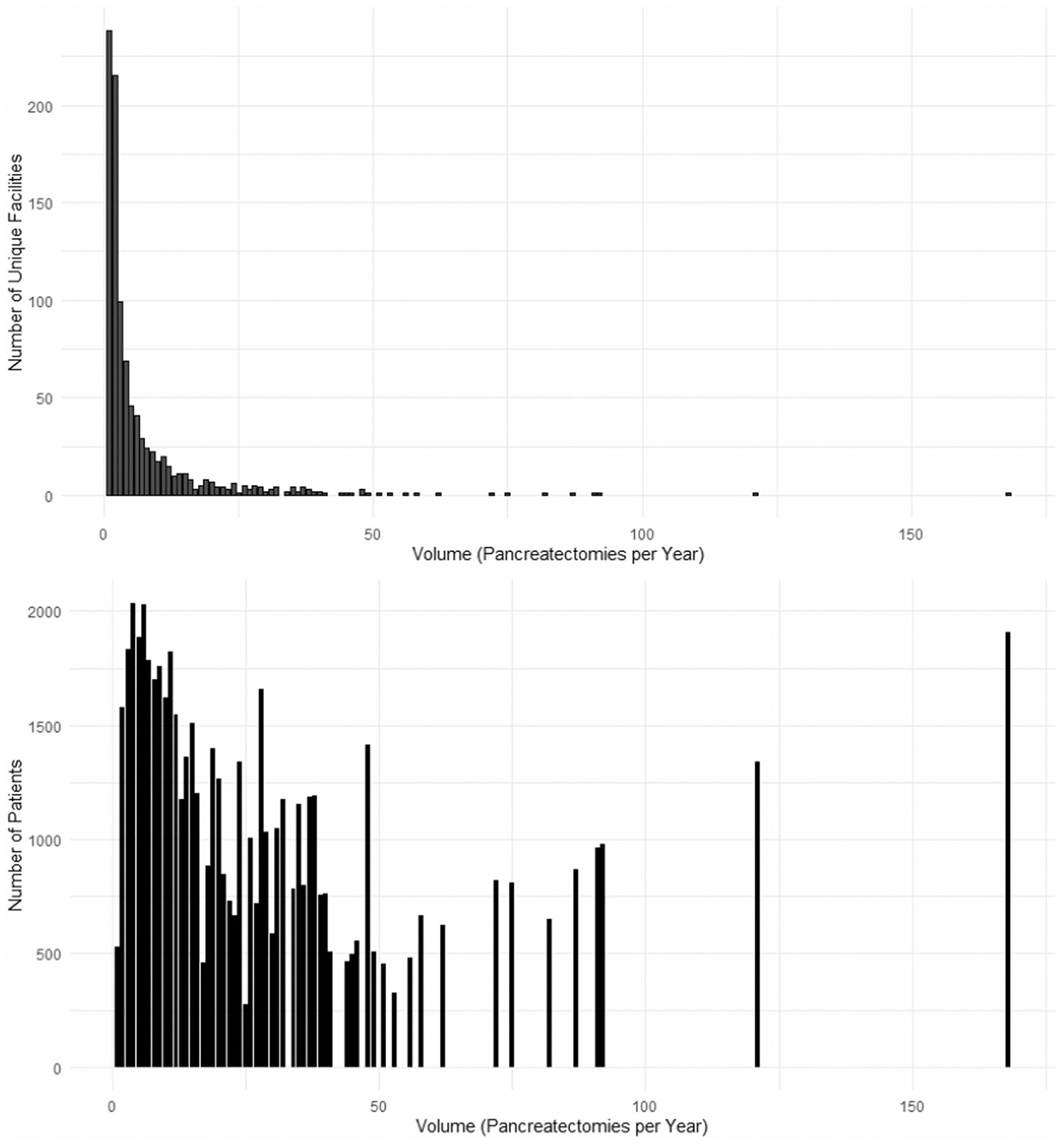
Number of patients and number of facilities per volume of pancreatectomies per year.

A total of 61,920 patients treated at 982 unique facilities were analyzed. Facility volume ranged from 1 to 168 pancreatectomies per year. Unique facilities are clustered in low volumes, whereas patients are more randomly distributed across all volumes. Facility and patient distribution are represented in Figure 1. Patients were slight majority male (51%) with an average age of 65 years old (SD ± 12), majority Non-Hispanic White (77%) and from a metropolitan area (85%) (Table 1). Patients had an overall 90-day mortality rate of 5.4% during the study period (2004–2021).

### 3.1 ∣ Quantiles and Comparison

Simple quantiles derived from number of patients revealed thresholds at < 9.9, 9.9–22.8, 22.9–39.9, > 39.9 (quartiles) or < 13.1, 13.1–34.8, > 34.8 (tertiles). These correspond to reductions in 90-day mortality across volume strata (quartiles: 8.3%, 5.8%, 4.2%, 3.4%; tertiles: 7.7%, 4.8%, 3.6%).

### 3.2 ∣ Restricted Cubic Spline (RCS)

From the optimized model (4 knots, locations at 5, 10, 30, and 40 cases per year), specific volume thresholds were identified (Optimal RCS model identification: Supporting Information Tables 2 and 3). The elbow method, taking the furthest point from the linear end point lines, yielded a cutoff of 26.1 cases per year. Thresholds identified by the point closest to (0, 1), Youden Index, and Index of Union were consistent at 19.5 cases per year. The inflection point (or second derivative) was identified at 9.2 cases per year. Overall, the range of threshold values produced was from 9.2 to 26.1 cases per year for RCS analysis. 90-day mortality was significantly different at each of the volume thresholds for the low-volume versus the high-volume group (Table 2).

### 3.3 ∣ Optimal Cutpoints

Within the CutpointR statistical package, the method that maximizes the metric function after LOESS smoothing yielded the lowest optimal cutpoint at 15.7 cases per year, followed by the method that maximizes the Youden-Index after kernel smoothing the distributions of the two classes at 18.4 cases per year. The highest optimal cutpoints were at the maximized Youden-Index parametrically at 45.5 cases per year and the sample mean as the optimal cutpoint at 33 cases per year. A total of 9 optimal cutpoints methods were used with an optimal cutoff ranging from 15.7 to 45.5 cases per year (Supporting Information Table 4). 90-day mortality was significantly different at each of the VTs for the low-volume versus the high-volume group (Table 2).

### 3.4 ∣ CART

Using the rpart statistical package, a CART was built based on the covariates age, sex, Charlson Deyo Score, race & ethnicity, insurance status, pathologic T stage, and facility volume (rounded to nearest integer). An initial complexity parameter was chosen of 0.0001. Initial splits or nodes in volume thresholds yielded 11 and 18 cases per year, depending on if your age was < 70.5 years old (Supporting Information Figure 2). Age carried the highest variable importance (40%), closely followed by volume (35%). All other covariates carried less than 10% variable importance (Supporting Information Table 5). To simplify or prune the CART, a complexity parameter of 0.00012 was chosen based on calculation of 1 standard error of the relative error. The first node or split where volume contributed to the model was at 18 cases per year. Again, age carried the highest variable importance (46%), followed by volume (32%). The remaining covariates carried an importance of less than 10%.

### 3.5 ∣ SSLR

Using the SSLR methodology, volume cutoffs were identified at 4, 10, 21, 48, and 121 cases per year. This yields 6 volume strata of ≤ 3, 4–9, 10–20, 21–47, 48–120, ≥ 121 cases per year. These cutoffs were determined based on nonoverlapping confidence intervals for the likelihood ratio of 90-day mortality (Supporting Information Table 6). 90-day mortality decreased across strata VTs (Table 3). A singular VT was identified at 21 pancreatectomies per year where the SSLR was < 1 for 90-day mortality. RCS, optimal cutpoints, and SSLR produced a singular VTs summarized in Table 2. Singular VTs ranged from 9 to 45. CART, quantiles, and SSLR produced multiple volume strata summarized in Table 3.

## 4 ∣ Discussion

Volume thresholds aim to identify hospitals that have improved patient outcomes (e.g. survival) for complex procedures, but volume threshold calculations in the literature to date have relied on varied statistical methods and models with often opaque statistical input parameters. This introduces potential for bias and has led to wide variability in the published threshold values [1, 5-7, 9, 10]. In the present analysis, we report variation between commonly used methodologies in derivation of both single volume thresholds and volume strata for the purposes of clinical research and quality reporting. When evaluating 90-day mortality after pancreatectomy with those methods, volume thresholds ranged from 9 to 45 cases per year, with variable single volume thresholds produced by RCS, Optimal Cutpoints, and CART statistical methods.

When using SSLR as the statistical method, there are no modifiable input or output parameters outside of the data itself, yielding a consistent and reproducible single cutoff. Moreover, SSLR produces multiple empirically discreet volume strata that may be more clinically distinct than traditional volume stratification methods such as quartile separation.

Past studies on pancreatic resections have relied on quantiles, RCS, and optimal cutpoints to define volume thresholds [25-29]. Although it is recognized that different study populations may give rise to different volume thresholds, what may be under-appreciated is that a single methodology can also produce variability in thresholds depending on modifiable model parameters. In the present study, VTs ranged from 9 to 45 with RCS and optimal cutpoints. Studies utilizing RCS have reported varying results. One study identified a volume cutoff of 20 procedures per year for minimally invasive pancreaticoduodenectomy using RCS. Another, analyzing all pancreatectomies, found a high-volume cutoff of 18 procedures per year despite also using RCS [26]. Additionally, national standards in volume thresholds in pancreatectomy, such as those established by the American Cancer Society and the Leapfrog Group, are largely based on expert consensus. Both organizations primarily reference two key studies: one is a systematic review that reports high volume cutoffs ranging from 5 to 90 cases per year, while the other employs quartiles to identify low and high-volume cutoffs ranging from 3.3 to 45 cases per year, respectively [5, 6]. Lastly, a study using tertiles derived from the National Inpatient Sample (NIS) demonstrated substantial changes in volume thresholds over an 11-year period. During this time, the low volume cutoff increased from 7 to 22 cases, while the high-volume cutoff increased from 29 to 99.5 cases [27]. This variability underscores methodological challenges in defining standard thresholds for surgical volumes in pancreatic cancer care. These historical results emphasize the significant influence that methodological choices can have on the resulting thresholds, corroborated by the variability observed in the current analysis.

In contrast to quantiles, RCS, and optimal cutpoints, the SSLR method produces multiple volume cutoffs without variability in input parameters, ensuring unique reproducibility of results. SSLR was originally introduced to identify optimal cutoffs in diagnostic tests such as the AUDIT-C and depression screenings [30, 31]. Since then, SSLR has been well documented in orthopedic surgery literature, particularly for total knee arthroplasty and total hip replacement procedures [22, 32-34]. In the context of pancreatectomies, SSLR identified 6 volume strata of ≤ 3, 4–9, 10–20, 21–47, 48–120, ≥ 121 cases per year. The ability of SSLR to yield multiple, empirically derived volume strata has several implications. First, this methodology may be superior to simple quantile stratification for multivariable modeling. For example, when defining hospital volume “groups” for modeling, SSLR-derived groups are more mathematically robust strata than simple tertiles or quartiles. Second, the multiple thresholds produced by SSLR may facilitate a more nuanced approach to volume-based assessments that inform healthcare assessing access, quality reporting, and policy [35-38]. With the access and quality implications of a policy-oriented threshold, it may be advantageous to publish a few strata for quality reporting, such as given with SSLR, rather than a single threshold. With a country as geographically expansive and socio-demographically diverse as the U.S., a tiered approach to characterizing surgical volume and outcomes may be advantageous in offering feasible tradeoffs between risk-reduction and costs of accessing “high-volume” providers. In scenarios where a single cutoff is required, SSLR can also be defined where the likelihood ratio crosses 1.0 for the strata [23, 31]. The singular cutoff derived from SSLR is potentially superior to other methodologies due to its lack of arbitrary input parameters, allowing for consistent and reproducible results across a particular data set.

This study has several limitations. First, the NCDB does not capture the diversity of facilities that deliver cancer care to pancreatic cancer patients in the U.S. population. Non-COC facilities, which are likely to have lower volumes, may be underrepresented, potentially underestimating the relationship between volume and outcomes within the data set. Although pancreatectomies are performed for noncancerous indications and the NCDB may underestimate true facility pancreatectomy volume, a recent study evaluating all pancreatomies at NSQIP hospitals showed that over 80% of pancreatectomies were performed for cancer [39]. Second, the present study utilized unadjusted analysis of optimal cutpoints and RCS to demonstrate consistency of input parameters across methodologies and not introduce another potential source of variability across methodologies. While this approach ensured comparability, it did not account for the variability introduced by multivariable regression models that could have been used in a more complex analysis. Additionally, random-effects or mixed-effects models to account for clustering of patients within facilities were not applied, which may influence the precision of variance estimates. Therefore, results of this study should only be construed as proof of concept. Third, the implementation of volume thresholds as policy presents challenges that are outside the scope of the present study. These challenges include ensuring appropriate, affordable, and timely access to care, the potential for hospital closure, and the logistical difficulties of centralizing high-volume procedures. Finally, any volume thresholds regardless of methodology will vary based on input data (e.g., NCDB, Medicare, etc). However, the authors believe that minimizing methodologic variability is still highly desirable even if individual thresholds may vary slightly across datasets.

## 5 ∣ Conclusion

Multiple methods exist for defining hospital-level procedural volume thresholds, including quantiles, restricted cubic splines, optimal cutpoints, classification and regression trees, and SSLR. The reproducibility of the SSLR methodology, along with its ability to identify multiple strata, may make it a desirable method for deriving empiric volume thresholds for quality reporting and research in health care. Its ability to provide robust, data-driven thresholds without reliance on arbitrary input parameters positions SSLR as a promising tool for informing development of future healthcare policy.

## Supplementary Material

Supplemental Table 5

Supplemental Table 6

Supplemental Table 4

Supplemental Table 3

Supplemental Table 2

Supplemental Table 1

Supplemental Figure 2

Supplemental Figure 1

Additional supporting information can be found online in the Supporting Information section.

**Supplemental Figure 1:** Elbow Method for Calculating Optimal Threshold in Cubic Splines. **Supplemental Figure 2:** Classification and Regression Tree (First few nodes/splits). **Supplemental Table 1:** Methodology used to determine thresholds – Variability in Input Parameters, Optimizing Model and then Output Methods of Interpretation. **Supplemental Table 2.** Default Locations for Cubic Spline Knots. **Supplemental Table 3:** Cubic Spline Input Parameters with Optimization of Knots. **Supplemental Table 4:** Summary of input parameters and outputs for CutpointR with 1000 bootstrap and the Youden index (Youden Index– statistical measure used to evaluate performance of a test). **Supplemental Table 5:** Classification and Regression Tree Variable Importance. Complexity parameter of 0.0001; Covariates age, sex, Charlson Deyo Score, Income class, race & ethnicity, insurance status, pathologic T stage, facility volume (rounded to nearest whole integer). **Supplemental Table 6:** SSLR outputs.

## Figures and Tables

**TABLE 1 ∣:** Cohort demographics.

Demographics	Overall *N* = 61,920
**Sex, male**	31,512 (51%)
**Age group**	
< 50	5956 (9.6%)
50–60	11,853 (19%)
60–70	20,274 (33%)
70–80	18,146 (29%)
80+	5691 (9.2%)
**Race ethnicity**	
Non-hispanic white	47,974 (77%)
Non-hispanic black	6278 (10%)
Hispanic	3502 (5.7%)
Asian	1834 (3.0%)
Other/unknown	2332 (3.8%)
**Metropolitan status**	
Metro	49,701 (85%)
Rural	950 (1.6%)
Urban	7660 (13%)
**Income quartile**	
Lowest Q1	8220 (15%)
Q2	11,057 (21%)
Q3	12,853 (24%)
Q4	21,427 (40%)
**Insurance status**	
Private	23,304 (38%)
Uninsured + Medicaid	4531 (7.3%)
Medicare	32,237 (52%)
Other/unknown	1848 (3.0%)
**Great circle distance**	
Median (IQR)	19 (8, 50)
**Charlson deyo score**	
0	39,336 (64%)
1	15,963 (26%)
2	4262 (6.9%)
3+	2359 (3.8%)
**Pancreatectomy type**	
Distal pancreatectomy	23,440 (38%)
Pancreaticoduodenectomy	38,480 (62%)

**TABLE 2 ∣:** Methodologies that produce a singular VT; *n* (%).

	Statistical method		Low VT, *n* (%)	High VT, *n* (%)
SSLR	1	Cutoffs	< 21	21+
		90 d Mortality	1961 (7.2%)	1163 (3.8%)
		Facilities	902 (92%)	80 (8%)
		Patients	30217 (49%)	31703 (51%)
RCS	1	Cutoffs	< 9.2	9.2+
		90 d Mortality	1159 (8.3%)	1965 (4.5%)
		Facilities	783 (80%)	199 (20%)
		Patients	15127 (24%)	46793 (76%)
	2–4	Cutoffs	< 19.5	19.5+
		90 d Mortality	1884 (7.2%)	1240 (3.9%)
		Facilities	891 (91%)	91 (9%)
		Patients	28103 (45%)	33817 (55%)
	5	Cutoffs	< 26.1	26.1+
		90 d Mortality	2138 (6.7%)	986 (3.8%)
		Facilities	921 (94%)	61 (6%)
		Patients	34234 (55%)	27686 (45%)
Optimal cutpoints	1	Cutoffs	< 15.7	15.7+
		90 d Mortality	1670 (7.5%)	1454 (4.1%)
		Facilities	867 (88%)	115 (12%)
		Patients	24157 (34%)	37763 (66%)
	2	Cutoffs	< 18.4	18.4+
		90 d Mortality	1804 (7.3%)	1320 (4.0%)
		Facilities	883 (90%)	99 (10%)
		Patients	26706 (43%)	35214 (57%)
	3–4	Cutoffs	< 19.5	19.5+
		90 d Mortality	1884 (7.2%)	1240 (3.9%)
		Facilities	891 (91%)	91 (9%)
		Patients	28103 (45%)	33817 (55%)
	5–6	Cutoffs	< 20.1	20.1+
		90 d Mortality	1961 (7.2%)	1163 (3.8%)
		Facilities	902 (92%)	80 (8%)
		Patients	30217 (49%)	31703 (51%)
	7	Cutoffs	< 22.8	22.8+
		90 d Mortality	2007 (7.0%)	1117 (3.8%)
		Facilities	906 (92%)	76 (8%)
		Patients	30948 (50%)	30972 (50%)
	8	Cutoffs	< 33.8	33.8+
		90 d Mortality	2387 (6.3%)	737 (3.6%)
		Facilities	942 (96%)	40 (4%)
		Patients	40457 (65%)	21463 (35%)
	9	Cutoffs	< 45.5	45.5+
		90 d Mortality	2725 (6.0%)	399 (3.1%)
		Facilities	964 (98%)	18 (2%)
		Patients	48554 (78%)	13366 (22%)

**TABLE 3 ∣:** Multiple strata methodologies; *n* (%) of facilities and patients within each representative volume strata.

Method	Volume strata						
CART	Cutoffs	<11	11–17	18+			
	90 d Mortality	1242 (8.0%)	519 (6.2%)	1363 (4.0%)			
	Facilities	800 (81%)	78 (8%)	104 (11%)			
	Patients	16746 (27%)	9074 (15%)	36100 (58%)			
SSLR	Cutoffs	<3	4–9	10–20	21–47	48–120	121+
	90 d Mortality	408 (11%)	751 (7.3%)	802 (6.1%)	787 (4.2%)	305 (3.3%)	71 (2.3%)
	Facilities	552 (56%)	231 (24%)	115 (12%)	67 (7%)	15 (2%)	2 (0.2%)
	Patients	3940 (6%)	11187 (18%)	14242 (23%)	19741 (32%)	9562 (15%)	3248 (5%)
Tertiles	Cutoffs	< 13.1	13.1–34.8	34.9+			
	90 d Mortality	1511 (7.7%)	907 (4.8%)	706 (3.6%)			
	Facilities	845 (86%)	99 (10%)	38 (4%)			
	Patients	21285 (34%)	19954 (32%)	20681 (33%)			
Quartiles	Cutoffs	< 9.9	9.9–22.8	22.9–39.9	40+		
	90 d Mortality	1159 (8.3%)	848 (5.8%)	612 (4.2%)	505 (3.4%)		
	Facilities	783 (80%)	123 (13%)	53 (5%)	23 (2%)		
	Patients	15127 (24%)	15821 (26%)	15373 (25%)	15599 (25%)		

## Data Availability

The data that support the findings of this study are available from American College of Surgeons Commission on Cancer. Restrictions apply to the availability of these data, which were used under license for this study. Data are available from the author(s) with the permission of American College of Surgeons Commission on Cancer.
