# Supplementary material for: Reproducibility and Consistency of Methods to Define Hospital-Level Procedural Volume Thresholds for Pancreatectomy
Source: J Surg Oncol. Author manuscript; Available in PMC 2026 Jul 25. (PMC13401272; doi:10.1002/jso.70134)
Supplement: Supplemental Table 5 [file NIHMS2190342-supplement-Supplemental_Table_5.docx]

Supplemental Table 5. Classification and Regression Tree Variable Importance. *Complexity parameter of 0.0001; Covariates age, sex, Charlson Deyo Score, Income class, race & ethnicity, insurance status, pathologic T stage, facility volume (rounded to nearest whole integer)*

| **Variable** | **Importance** | **Percentage** |
| --- | --- | --- |
| Age | 180 | 40% |
| Volume | 159 | 35% |
| Insurance | 39 | 8.8% |
| Path T Stage | 35 | 7.7% |
| Race/Ethnicity | 11 | 2.5% |
| Charlson-Deyo Score | 9.5 | 2.1% |
| Income | 8.8 | 2.0% |
| Sex | 5.8 | 1.3% |
