# Supplementary material for: Reproducibility and Consistency of Methods to Define Hospital-Level Procedural Volume Thresholds for Pancreatectomy
Source: J Surg Oncol. Author manuscript; Available in PMC 2026 Jul 25. (PMC13401272; doi:10.1002/jso.70134)
Supplement: Supplemental Table 6 [file NIHMS2190342-supplement-Supplemental_Table_6.docx]

Supplemental Table 6. SSLR outputs

*SSLR – Likelihood ratio of 90-day mortality within that volume threshold*

*CI – Confidence intervals based on the variance of the SSLR*

*Methodology – Starting with whole number volume strata (e.g. 1, 2, 3, 4, etc) SSLR calculated. CI Low is compared for first strata to CI high of second strata. If overlap in these CI, they are combined, the SSLR is recalculated and sequentially compared to the next volume strata. If they do not overlap, the next volume strata is used as the next comparison with the sequential volume strata. Resulting volume strata are displayed following collapse of CI for this model.*

| **Volume** | **SSLR** | **CI Low** | **CI High** |
| --- | --- | --- | --- |
| 1 to 3 | 2.18 | 1.98 | 2.40 |
| 4 to 9 | 1.39 | 1.30 | 1.48 |
| 10 to 20 | 1.14 | 1.07 | 1.21 |
| 21 to 47 | 0.78 | 0.73 | 0.82 |
| 48 to 120 | 0.61 | 0.55 | 0.68 |
| 121+ | 0.41 | 0.32 | 0.51 |
