# Supplementary material for: Reproducibility and Consistency of Methods to Define Hospital-Level Procedural Volume Thresholds for Pancreatectomy
Source: J Surg Oncol. Author manuscript; Available in PMC 2026 Jul 25. (PMC13401272; doi:10.1002/jso.70134)
Supplement: Supplemental Table 4 [file NIHMS2190342-supplement-Supplemental_Table_4.docx]

Supplemental Table 4. Summary of input parameters and outputs for CutpointR with 1000 bootstrap and the Youden index (Youden Index– statistical measure used to evaluate performance of a test)

| **Method** |  | **Optimal Cutpoint** |
| --- | --- | --- |
| maximize_metric | Maximize the metric function | 20.3 |
| maximize_spline_metric | Maximize the metric function after spline smoothing | 19.7 |
| maximize_loess_metric | Maximize the metric function after LOESS smoothing | 15.7 |
| maximize_boot_metric | Bootstrap the optimal cutpoint when maximizing a metric | 20.1 |
| maximize_gam_metric | Maximize the metric function after smoothing via Generalized Additive Models | 19.5 |
| oc_youden_kernel | Maximize the Youden-Index after kernel smoothing the distributions of the two classes | 18.4 |
| oc_mean | Use the sample mean as the optimal cutpoint | 33.8 |
| oc_median | Use the sample median as the optimal cutpoint | 22.8 |
| oc_youden_normal | Maximize the Youden-Index parametrically | 45.5 |
