# Supplementary material for: Reproducibility and Consistency of Methods to Define Hospital-Level Procedural Volume Thresholds for Pancreatectomy
Source: J Surg Oncol. Author manuscript; Available in PMC 2026 Jul 25. (PMC13401272; doi:10.1002/jso.70134)
Supplement: Supplemental Table 3 [file NIHMS2190342-supplement-Supplemental_Table_3.docx]

Supplemental Table 3. Cubic Spline Input Parameters with Optimization of Knots

*3a. RCS with variable number of knots. Optimal model at 4 knots (bold)*

*3b. RCS with variable knot locations. Optimal model at locations 5, 10, 30, 40 (bold)*

| 1. **Testing Knots** | | | | |
| --- | --- | --- | --- | --- |
| **Knots** | 3 | **4** | 5 | 6 |
| **AIC** | 23952.03 | **23933.5** | 23934.44 | 23933.3 |
| **BIC** | 23978.94 | **23969.39** | 23979.3 | 23987.13 |
| 1. **Testing Location** | | | | |
| **Knots** | 4 | 4 | **4** | 4 |
| **Location 1** | 3.07143 | 10 | **5** | 5 |
| **Location 2** | 14.17647 | 20 | **10** | 10 |
| **Location 3** | 33.8889 | 30 | **30** | 40 |
| **Location 4** | 121.27778 | 40 | **40** | 80 |
| **AIC** | 23933.5 | 23936.51 | **23933.41** | 23933.63 |
| **BIC** | 23969.39 | 23972.4 | **23969.3** | 23969.52 |
