# Supplementary material for: Reproducibility and Consistency of Methods to Define Hospital-Level Procedural Volume Thresholds for Pancreatectomy
Source: J Surg Oncol. Author manuscript; Available in PMC 2026 Jul 25. (PMC13401272; doi:10.1002/jso.70134)
Supplement: Supplemental Table 1 [file NIHMS2190342-supplement-Supplemental_Table_1.docx]

Supplemental Table 1. Methodology used to determine thresholds – Variability in Input Parameters, Optimizing Model and then Output Methods of Interpretation

| **Method** | **Cubic Splines** | **Optimal cutpoints** | **Classification & Regression Trees** | **SSLR** |
| --- | --- | --- | --- | --- |
| **Input Parameters** | Number of Knots  Knot Locations | Methodology Metric | Covariates  Complexity Parameter | Volume Strata Granularity |
| **Optimize Input Parameters** | Minimizing AIC and BIC | Bootstrap | Not applicable | Not applicable |
| **Output Methods (Number of Thresholds)** | Elbow Method (1)  Youden (1)  Point (0,1)  Index of Unionization  Inflection (max of 2^nd^ derivative) | Change point (1) | Selection of particular Node (Multiple) | Thresholds without overlapping confidence intervals (Multiple) |
