# Supplementary material for: Reproducibility and Consistency of Methods to Define Hospital-Level Procedural Volume Thresholds for Pancreatectomy
Source: J Surg Oncol. Author manuscript; Available in PMC 2026 Jul 25. (PMC13401272; doi:10.1002/jso.70134)
Supplement: Supplemental Figure 2 [file NIHMS2190342-supplement-Supplemental_Figure_2.docx]

Supplemental Figure 2. Classification and Regression Tree (First few nodes/splits). *Any movement to the Right is Yes; Movement Left is No;* *Complexity parameter of 0.0001; Covariates age, sex, Charlson Deyo Score, Income class, race & ethnicity, insurance status, pathologic T stage, facility volume (rounded to nearest whole integer)*
