# Supplementary material for: Reproducibility and Consistency of Methods to Define Hospital-Level Procedural Volume Thresholds for Pancreatectomy
Source: J Surg Oncol. Author manuscript; Available in PMC 2026 Jul 25. (PMC13401272; doi:10.1002/jso.70134)

Supplemental Figure 1. Elbow Method for Calculating Optimal Threshold in Cubic Splines

*Blue line: cubic spline model based on 4 knots at default locations*

*Red dashed line: Using the endpoints of cubic spline, line drawn between two points*

*Dot: Furthest point (and optimized threshold) from the linear end point line (e.g. the elbow)*


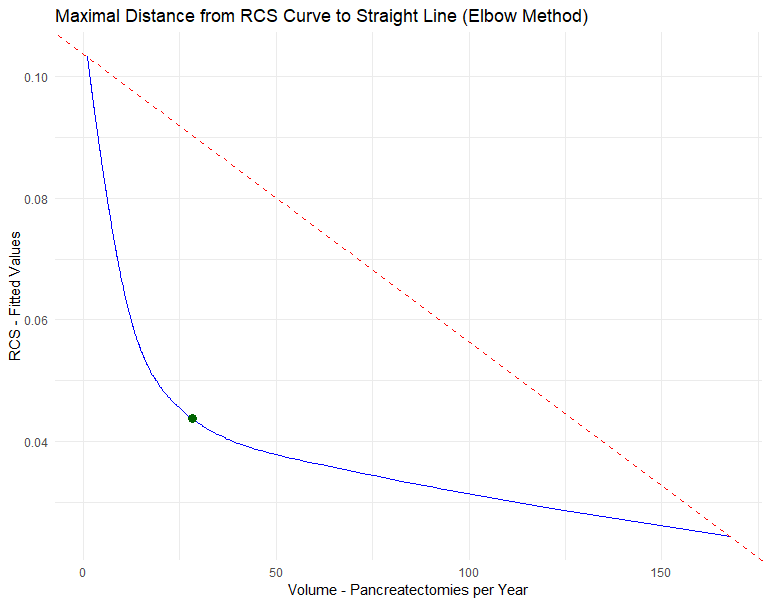

Supplement: Supplemental Figure 1 [file NIHMS2190342-supplement-Supplemental_Figure_1.docx]
