# Supplementary material for: Reproducibility and Consistency of Methods to Define Hospital-Level Procedural Volume Thresholds for Pancreatectomy
Source: J Surg Oncol. Author manuscript; Available in PMC 2026 Jul 25. (PMC13401272; doi:10.1002/jso.70134)
Supplement: Supplementary file 8 [file NIHMS2190342-supplement-Supplemental_Table_2.docx]

Supplemental Table 2. Default Locations for Cubic Spline Knots[14]

Default locations placed at quantiles of X based on number of knots input into cubic splines. Knots are the independent locations on the curve where the transition or change in behavior of the curve is allowed. Knots ensure smooth overall curve.

Number of Knots	Knot Locations (quantiles of x variable)					
3	0.1	0.5	0.9			
4	0.05	0.35	0.65	0.95		
5	0.05	0.275	0.5	0.725	0.95	
6	0.05	0.23	0.41	0.59	0.77	0.95
